# Resistance to Paclitaxel in a Cisplatin-Resistant Ovarian Cancer Cell Line Is Mediated by P-Glycoprotein

**DOI:** 10.1371/journal.pone.0040717

**Published:** 2012-07-11

**Authors:** Britta Stordal, Marion Hamon, Victoria McEneaney, Sandra Roche, Jean-Pierre Gillet, John J. O’Leary, Michael Gottesman, Martin Clynes

**Affiliations:** 1 National Institute for Cellular Biotechnology, Dublin City University, Dublin, Ireland; 2 Department of Histopathology, St James’ Hospital and Trinity College Dublin, Dublin, Ireland; 3 Laboratory of Cell Biology, National Cancer Institute, National Institute of Health, Bethesda, Maryland, United States of America; University of Texas Health Science Center at San Antonio, United States of America

## Abstract

The IGROVCDDP cisplatin-resistant ovarian cancer cell line is also resistant to paclitaxel and models the resistance phenotype of relapsed ovarian cancer patients after first-line platinum/taxane chemotherapy. A TaqMan low-density array (TLDA) was used to characterise the expression of 380 genes associated with chemotherapy resistance in IGROVCDDP cells. Paclitaxel resistance in IGROVCDDP is mediated by gene and protein overexpression of P-glycoprotein and the protein is functionally active. Cisplatin resistance was not reversed by elacridar, confirming that cisplatin is not a P-glycoprotein substrate. Cisplatin resistance in IGROVCDDP is multifactorial and is mediated in part by the glutathione pathway and decreased accumulation of drug. Total cellular glutathione was not increased. However, the enzyme activity of GSR and GGT1 were up-regulated. The cellular localisation of copper transporter CTR1 changed from membrane associated in IGROV-1 to cytoplasmic in IGROVCDDP. This may mediate the previously reported accumulation defect. There was decreased expression of the sodium potassium pump (ATP1A), MRP1 and FBP which all have been previously associated with platinum accumulation defects in platinum-resistant cell lines. Cellular localisation of MRP1 was also altered in IGROVCDDP shifting basolaterally, compared to IGROV-1. BRCA1 was also up-regulated at the gene and protein level. The overexpression of P-glycoprotein in a resistant model developed with cisplatin is unusual. This demonstrates that P-glycoprotein can be up-regulated as a generalised stress response rather than as a specific response to a substrate. Mechanisms characterised in IGROVCDDP cells may be applicable to relapsed ovarian cancer patients treated with frontline platinum/taxane chemotherapy.

## Introduction

The prognosis for women with ovarian cancer is very poor. The majority of patients present with advanced disease and the long-term survival in these patients is 10–30% [1]. Current treatment of ovarian cancer is surgery followed by platinum/taxane combination chemotherapy [1]. The chemotherapeutic drugs cisplatin and paclitaxel are used in the treatment of many solid tumours, including ovarian carcinoma. Cisplatin binds to the DNA strand, hindering both DNA replication and RNA translation and eventually triggering apoptosis. Paclitaxel causes cytotoxicity by binding to and stabilising polymerised microtubules. Due to their differing mechanisms of action, platinums and taxanes are often combined in cancer therapy. Initial responsiveness to chemotherapy in ovarian cancer is high, but up to 80% of patients will eventually relapse and become platinum/taxane resistant.

The IGROVCDDP cisplatin-resistant ovarian cell line is an unusual cisplatin-resistant model, as it is also cross-resistant to paclitaxel. When acquired cisplatin resistance is produced in cell lines, only 17% are also resistant to paclitaxel [2]. 41% of cisplatin drug-resistant models are not resistant to paclitaxel and 28% of cell models become hypersensitive to paclitaxel [2]. This suggests that the majority of cancer patients would benefit from receiving chemotherapy which alternates between cisplatin and paclitaxel, as developing resistance to one drug is less likely to result in resistance to the other. The challenge is how to identify which patients will respond well to alternating therapy between cisplatin and paclitaxel. This is because while the majority of cancer patients may respond well to this treatment strategy, the cross resistant cohort, would respond poorly and need to be treated with alternate therapy.

IGROVCDDP models the resistance phenotype of ovarian cancer patients who have failed standard frontline combination platinum/taxane chemotherapy. Chemotherapeutic drugs which IGROVCDDP is sensitive to may be suitable for the treatment of platinum/taxane resistant ovarian cancer. Studying the IGROVCDDP drug-resistant model will allow us to understand the mechanisms of cross resistance between platinums and taxanes. It is our aim to translate molecular markers of this cross resistance phenotypes to the clinical treatment of relapsed drug-resistant ovarian carcinoma.

## Methods

### Cell Culture and Cytotoxicity Assays

The human IGROV-1 ovarian cancer cell line and its cisplatin-resistant variant IGROVCDDP were obtained from Prof. Jan Schellens [3], [4]. Cells were grown in antibiotic and chemotherapy-free RPMI (Sigma #R8758) with 10% FCS (Lonza, Belgium). Cells were maintained in a humidified atmosphere with 5% CO2 at 37°C, and were *mycoplasma-*free. To determine cytotoxicity cells (1×10^4^ cells/well) were plated into flat-bottomed, 96-well plates and allowed to attach overnight. Wells were treated in triplicate with serial dilutions of drug in a final volume of 200 µL. Drug-free controls were included in each assay. Plates were incubated for a further 5 days and cell viability was determined using an acid phosphatase assay [5].

### TaqMan Low Density Array (TLDA)

Cells (1.25×10^6^ cells/10 cm dish) were plated and allowed to attach and grow for 3 days to reach 70–80% confluence. The cells were then trypsinised, washed in 10 mL PBS, centrifuged and the supernatant removed. The cell pellets were stored at −80°C prior to analysis. Total RNA was prepared using a RNeasy Mini Kit (Qiagen, UK). The TLDA array was performed on biological triplicate samples as described in Gillet *et al*. 2011 [6]. The median expression of each sample was subtracted from all gene expressions for that sample. The data was analysed using *BRB ArrayTools*, a microarray-data statistical analysis tool (http://linus.nci.nih.gov/BRB-ArrayTools.html) [7]. Genes expressed by less than 50% of the samples were filtered out and a univariate two-sample T-test was performed to determine genes that were significantly different between IGROV-1 and IGROVCDDP based on a p<0.01 cutoff.

### Epirubicin Accumulation Assay

Cells were plated at a density of 2.5×10^5^ in a non-vented T25 flask. The next day the media was removed and the cells were treated with 1 µM epirubicin for 2 hours in the presence or absence of 0.25 µM elacridar, 0.67 µM, 3.33 µM or 33.3 µM cisplatin. Cells were washed with 4 mL of cold PBS and trypsinised. The cells were centrifuged and resuspended in 1 mL of PBS and a cell count performed (9 µL). The remaining cells were centrifuged, supernatant removed and the pellet stored at −20°C prior to analysis. Total epirubicin was then quantified by LC-MS following a liquid-liquid extraction sample preparation, according to the method of Wall *et al*. 2007 [8].

### Total Cellular Glutathione Assay

Cells were plated at a density of 1.25×10^6^ cells in a 10 cm diameter dish and allowed to attach overnight. The cells were drug treated for 24 hours, then trypsinised and a cell count performed. The cells were washed in 10 mL PBS, centrifuged and the supernatant removed. The cell pellets were stored at −20°C prior to analysis. Total glutathione was determined using a modification of Suzakake *et al*. [9]. Cell pellets were lysed in 150 µL water and sonicated, 12.5 µL of 30% sulfosalicyclic acid was added and the samples were vortexed. After 30 minutes on ice, protein-free supernatants were collected by centrifugation (12000 *g* for 5 minutes at 4°C). Glutathione concentration was determined using a reaction mixture containing 20 µL of lysate or standard, 90 µL of triethanolamine buffer, pH 8.0 (0.2 M), 30 µL of NADPH (4 mM) and 20 uL of DTNB (6 mM). After 2 minutes at 30°C, the reaction was started by the addition of 0.3 units of glutathione reductase per well. The plates were read at 405 nM (preheating to 30°C) with kinetic measurement by a plate reader synergy HT, Bio-Tek® (MASON Technology). The rate of change of the kinetic assay was then calculated by KC4 software.

### Glutathione Reductase (GSR) and Gamma Glutamyl Transpeptidase (GGT1) Enzyme Assays

Cell culture - Cells (6.25×10^5^ cells/10 cm dish) were plated and allowed to attach overnight. Cells were then treated with 0.67 µM cisplatin. Drug-treated cells and their controls were trypsinised and a cell count performed. The cells were then washed in 10 mL PBS, centrifuged and the supernatant removed. The pellet was resuspended in 400 µL cold enzyme assay buffer (100 mM potassium phosphate monobasic, 100 mM EDTA; pH 7.5). 16 µL of 25× Complete Protease Inhibitor (Roche, UK) was added, and the sample was sonicated. After centrifugation (13000 rpm for 15 minutes at 4°C) the supernatant was collected and frozen at −80°C prior to analysis.

GSR – GSR (Sigma) standards were made up in glutathione reductase dilution buffer (100 mM potassium phosphate monobasic; 100 mM EDTA; 1 mg/mL BSA; pH 7.5) ranging from 0.3–0.0037 units/mL. 40 µL of each sample and standard were assayed in duplicate in 96 well plates. The reaction mix was then added 160 µL total volume (2 mM oxidised glutathione (100 µL); 3 mM DNTB (50 µL); 2 mM NADPH (10 µL)).

GGT1**–** This method was adapted from the method of Silber *et al*. [10]. GGT1 (Patricell, UK) standards were made up in water ranging from 1000–1.6 units/L. 30 µL of each sample and standard was assayed in duplicate in 96 well plates. 100 µL reaction mix was then added (60 mM gamma-glutamyl-p-nitroalinine (10 µL); 55.5 mM glyclglycine in 133.33 mM Tris Base pH 8.5 (90 µL)).

Analysis - The plates were read at 412 nM (preheating to 30°C) and 405 nM (preheating to 37°C) for GSR and GGT1 respectively, with kinetic measurement by a plate reader as described for the glutathione assay.

### Western Blots

Cells (1.25×10^6^ cells/10 cm dish) were plated and allowed to attach overnight. The cells were then drug-treated with cisplatin and grown for 3 days. Cells were resuspended in 100 µL lysis buffer (0.01 M Tris/HCl, pH 7.4) and sonicated. 20 µg of protein was diluted in Laemmli sample buffer, boiled for 3 minutes, cooled on ice and loaded onto 12% Tris/glycine gels with a 4% stacking gel. Samples and molecular weight markers were then electrophoresised for 90 minutes at 100 V. The gels were electrotransferred to 0.45 µm nitrocellulose membranes (Biorad) for 90 minutes at 100 V using a wet transfer system (Biorad). The membranes were blocked with 5% non-fat skim milk (Biorad) in PBS for 2 hours, then incubated with the primary antibody prepared in 3% skim milk/0.1% tween/PBS (Table 1) overnight at 4°C [11]. The membranes were washed in 0.3% tween/PBS 3×10 minutes and then incubated for 1 hour with a HRP-conjugated secondary antibody (Table 1). Membranes were washed again and exposed to luminol reagent (Santa Cruz) or ECL advanced western blotting reagent (GE Healthcare). Membranes were then exposed to autoradiographic film. β-actin blots were developed using an alkaline phosphatase antibody (Table 1) and Sigma Fast BICP reagent. Densitometry on a minimum of n = 3 biological replicates was performed using Quantity One software (Biorad), using local background correction. Abundance of protein was normalised to ponceau for each sample and then each biological series was normalised to IGROV-1.

**Table 1:** Antibodies for western blotting and confocal microscopy.

Protein	kDa	Host	Supplier	Catalogue #	Dilution Western	Dilution Confocal
ATP1A1	110	Mouse	Abcam	ab2872	1∶250	N/A
ATP7A	180	Rabbit	Gift from Prof. Anthony Monaco as described [11]		1∶1000	N/A
BCRP	72	Mouse	Alexis	ALX-801-029-C250	1∶250	N/A
BRCA1	220	Rabbit	Cell Signalling Technology	9010	1∶500	N/A
β-Actin	42	Mouse	Sigma	A5441	1∶10,000	N/A
CTR1/SLC31A1	30	Rabbit	Novus	NB100-402	1∶1000	1∶250
FBP	N/A	Rabbit	Novus	NBP1-32293	N/A	1∶250
GM130	N/A	Mouse	Transduction Labs	610823	N/A	1∶500
GCLC (γGCS)	73	Mouse	Abcam	ab55435	1∶500	N/A
GGT1	61.4	Mouse	Sigma	WH0002678M1-100UG	1∶1000	N/A
GSR	56.2	Mouse	Sigma	WH0002936M1-100UG	1∶1000	N/A
MRP1	190	Rat	Alexis	ALX-801-007-C250	1∶250	1∶250
MRP2	180	Mouse	Alexis	ALX-801-016-C250	1∶250	N/A
P-glycoprotein	170	Mouse	Alexis	ALX-801-002-C100	1∶250	N/A
Anti-Mouse HRP	N/A	Sheep	Sigma	A6782	1∶1000	N/A
Anti-Rabbit HRP	N/A	Goat	Sigma	A4914	1∶1000	N/A
Anti-Mouse AP	N/A	Rabbit	Sigma	A4312	1∶1000	N/A
Anit-Rat Alexa488	N/A	Donkey	Invitrogen	A21208	N/A	1∶500
Anti-Rabbit Alexa488	N/A	Goat	Invitrogen	A11008	N/A	1∶500
Anti-Mouse Alexa594	N/A	Goat	Invitrogen	A11005	N/A	1∶500

AP – Alkaline Phosphatase, ATP1A1 - Na^+^/K^+^ transporting alpha 1, ATP7A - ATPase, Cu++ transporting, alpha polypeptide, BCRP - Breast Cancer Resistance Protein, BRCA1 - Breast Cancer Susceptibility Protein 1, CTR1 - solute carrier family 31 (copper transporters), member 1, FBP – Folate Binding Protein, GM130 - Golgin A2, γGCS – gamma Glutamyl Cysteine Synthesase, GSR - Glutathione Reductase, GGT1 - Gamma Glutamyl Transpeptidase, HRP – Horseradish Peroxidase, MRP1 - Multidrug resistance-associated protein-1, MRP2 - Multidrug resistance-associated protein-2.

### Confocal Microscopy

Cells (1.5×10^5^ cells/well) were plated into 8-well chamber slides and allowed to attach overnight. All washes were with PBS and all incubations were at room temperature unless otherwise specified. The cells were washed twice, and fixed with 4% paraformaldehyde (Sigma) in PBS for 30 minutes at 37°C. The cells were permabilised with 0.5% Triton-X-100 (Sigma) for 10 minutes and washed twice. Cells were stained with a 50 µg/mL fluorescent TRITC solution in PBS for 40 minutes and then washed twice. The cells were then incubated with blocking buffer (0.02% BSA in PBS) for 30 minutes at 37°C. The cells were then incubated with primary antibody (Table 1) for 2 hours in a humidified atmosphere. The cells were then washed 3 times for 5 minutes and incubated with secondary antibody (Table 1) for 1 hour. The cells were then washed 3 times for 5 minutes. The cells were then coverslipped using mounting media containing DAPI (Sigma) and stored at 4°C before microscopy. Images were captured at x63 magnification and ×1 zoom. Scans were performed at 1 µm interval depths through the fixed cells, and single or merged images are presented either as XY single planes through the mid-section of the cells or orthogonal view.

### Statistical Analysis

All experiments were performed at minimum in triplicate. Two-sample, two tailed student’s t-tests were used to determine significant differences using p<0.05 as a cut off.

## Results

### Taxane Resistance in IGROVCDDP is Mediated by P-glycoprotein

The IGROV-1 and IGROVCDDP cells were analysed for 380 genes associated with chemoresistance by TLDA array in order to characterise the mechanisms of platinum and taxane resistance. 145 genes were found to be significantly different between IGROV-1 and IGROVCDDP based on a p<0.01 cutoff. Genes chosen for further analyses were based on the most significant by p-value as well as those pathways previously associated with platinum and taxane resistance (Table 2). All genes listed in table two were validated at the protein level by western blot.

**Figure 1:**
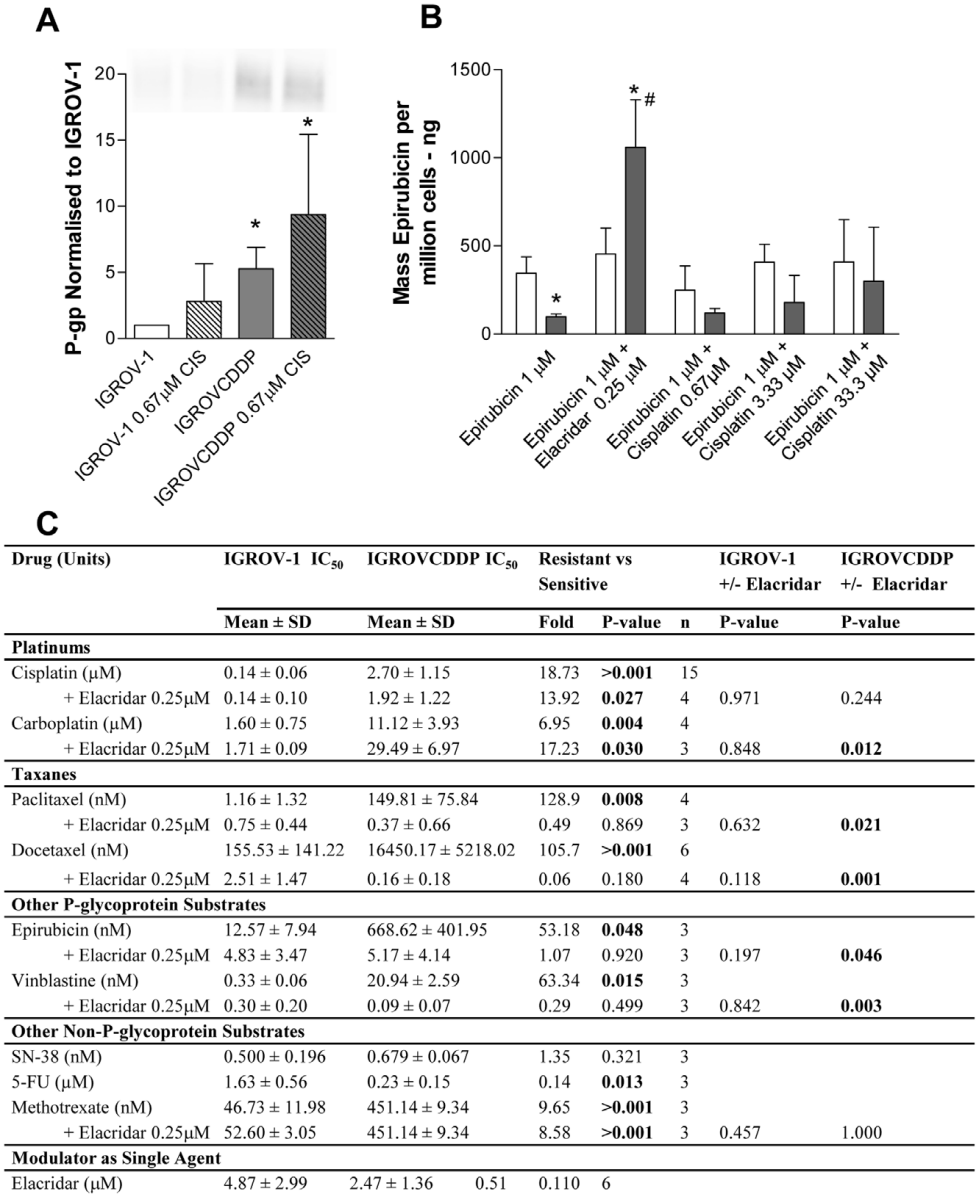
P-gp in IGROV-1 and IGROVCDDP cells. A) Western blot of P-glycoprotein, IGROV-1 (open bars) and IGROVCDDP (grey bars) with and without treatment with 0.67 µM cisplatin for 72 hours (striped bars). Representative image shown. Graph shows quantitation of n = 6 biological repeats normalised to β-actin. * Indicates significant difference from IGROV-1 p<0.05 student’s t-test. B) Accumulation of epirubicin determined by LC-MS. IGROV-1 (open bars) and IGROVCDDP (shaded bars). Cells were treated with 1 µM epirubicin for 2 hours, 0.25 µM elacridar, 0.67 µM, 3.33 µM or 33.3 µM cisplatin were investigated as modulators of epirubicin accumulation. Graph shows quantitation of n = 3 biological repeats normalised to cell number. * Indicates a significant difference between IGROV-1 and IGROV-CDDP, # Indicates a significant difference on the addition of a modulator (p<0.05 students t-test). C) Cytotoxicity of IGROV-1 and IGROVCDDP to P-glycoprotein and non P-glycoprotein substrates.

The gene expression of P-glycoprotein (P-gp) is increased in IGROVCDDP (Table 2), and there is a corresponding increase in protein expression (Figure 1A). A 3-day treatment with low dose cisplatin tended to increase P-gp expression in both IGROV-1 and IGROVCDDP but this was not significant (Figure 1A). P-gp was also confirmed to be functionally active in IGROVCDDP with an epirubicin accumulation assay (Figure 1B). The IGROVCDDP cells have significantly lower levels of P-gp substrate epirubicin after a 2-hour exposure compared to IGROV-1. When IGROVCDDP was treated with 0.25 µM of the P-gp inhibitor elacridar, which prevents the action of the drug pump [12] the accumulated mass of epirubicin increased and was significantly higher than that of the parent IGROV-1 cells. The increase above the level of IGROV-1 is an interesting observation, and may be due to the IGROVCDDP cells being so dependent on P-glycoprotein for drug efflux; they suffer more accumulation of drug when it is inhibited.

**Table 2:** TLDA IGROV-1 vs IGROVCDDP - Genes of interest by function or pathway.

Gene	Full Name/Synonyms			Mean mRNA Fold Change	SD	P-value
**Transporters not associated with platinum resistance**						
ABCB1		P-glycoprotein	**↑**	11.38	0.45	2.29E–06
ABCG2		BCRP/Breast Cancer Resistance Protein	**↓**	−2.17	0.19	2.95E–02
**Transporters which can directly efflux platinum**						
ABCC2		MRP2 cMOAT	**↓**	−3.27	0.07	1.15E–03
**Transporters that do not directly efflux platinum that are potential biomarkers of platinum accumulation defects**						
ATP1A1		Na^+^/K^+^ transporting alpha 1	**↓**	−4.52	0.02	1.09E–05
ABCC1		MRP1	**↓**	−1.43	0.02	3.15E–04
**Glutathione Metabolism**						
GSR		Glutathione Reductase	**↑**	1.40	0.12	1.24E–02
GGT1		Gamma Glutamyl Transpeptidase	**↑**	4.92	1.75	5.90E–04
**DNA Repair**						
BRCA1		Breast Cancer Susceptibility Protein 1	**↑**	2.17	0.25	1.31E–03

IGROVCDDP cells were screened for their response to a variety of chemotherapeutics (Figure 1C). IGROVCDDP cells are significantly resistant to non-P-gp substrates cisplatin and carboplatin [13]. IGROVCDDP is also significantly resistant to P-gp substrates [14]; taxanes, paclitaxel and taxotere, the anthracycline epirubicin and vinca alkaloid vinblastine. In contrast, IGROVCDDP is hypersensitive to treatment with non-P-gp substrate 5-FU [15]. IGROVCDDP is resistant to MRP1 substrate methotrexate [14] but not resistant to BCRP substrate SN-38 (Figure 1C) [16]. Treatment with 0.25 µM elacridar significantly reverses the resistance of the IGROVCDDP cells to all the P-gp substrates, but not the resistance to cisplatin, carboplatin and methotrexate. IGROVCDDP cells are also more sensitive to elacridar treatment than IGROV-1 (Table 3). IGROVCDDP cells have decreased mRNA expression of BCRP (Table 2) and it is not detectable by western blot (data not shown). This suggests that the reversal effects seen with elacridar treatment are specific to P-gp and not BCRP, which elacridar also inhibits.

**Table 3:** Resistance profile of IGROVCDDP to cisplatin and ouabain.

Drug (Units)	IGROV-1 IC_50_	IGROVCDDP IC_50_	Resistant vs Sensitive			IGROV-1+/− Ouabain	IGROVCDDP +/− Ouabain
	Mean ± SD	Mean ± SD	Fold	P-value	n	P-value	P-value
**Platinums**							
Cisplatin (µM)	0.14±0.06	2.70±1.15	18.73	**>0.001**	15		
+ Ouabain 0.01 nM	0.07±0.00	1.47±0.72	21.69	**0.027**	3	0.226	0.073
**Modulator as Single Agent**							
Ouabain (nM)	23.84±9.11	3.38±0.59	0.14	**0.018**	3		

The impact of co- or pre-treatment with cisplatin on paclitaxel cytotoxicity was investigated and no significant change was observed (data not shown). Similarly, co- or pre-treatment with paclitaxel did not reverse cisplatin resistance (data not shown).

Platinum resistance is associated with an intracellular shift of platinum uptake transporter CTR1 not resistance mediated by MRP2.

**Figure 2:**
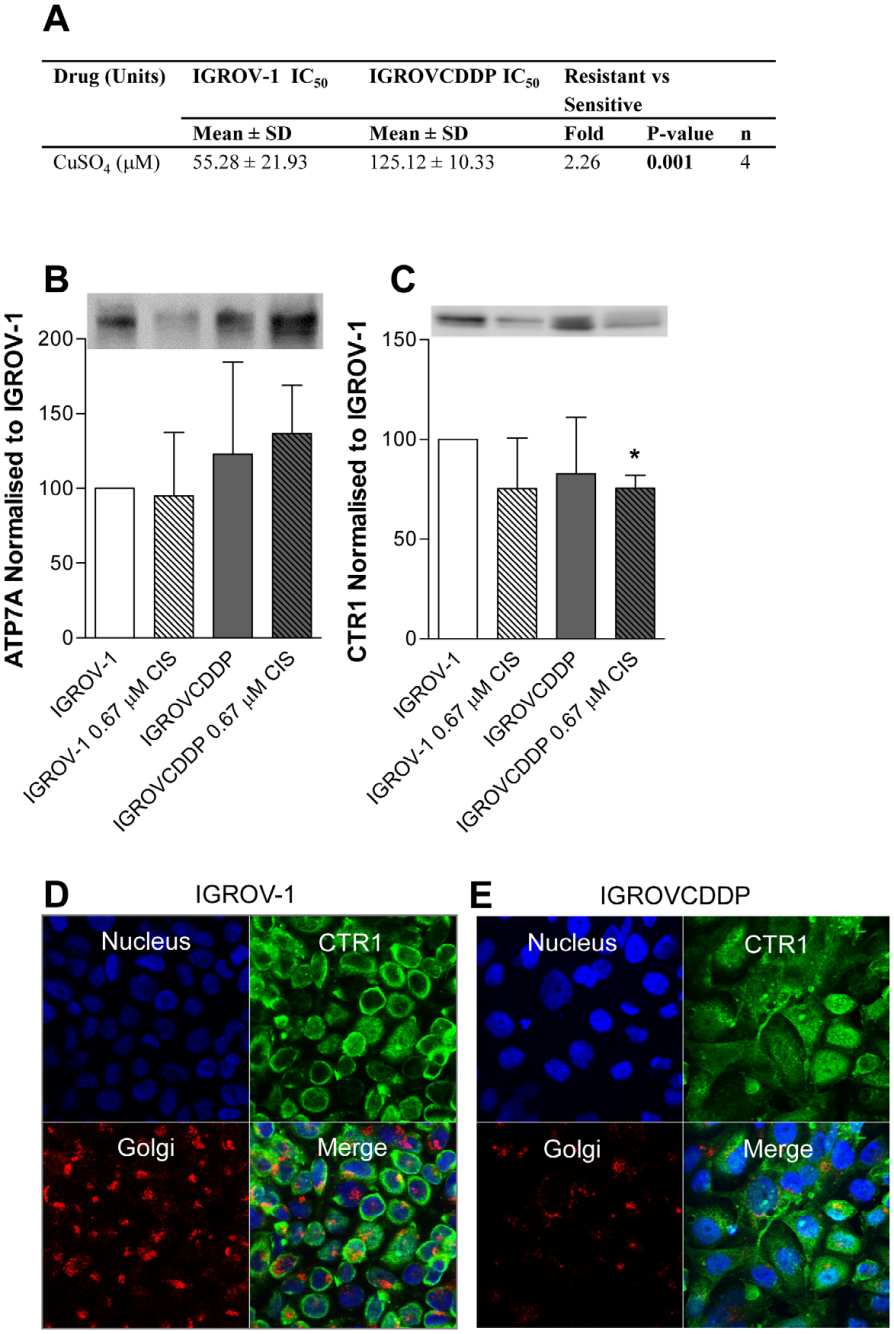
Copper Transporters in IGROV-1 and IGROVCDDP cells. A) Cytotoxicity of IGROV-1 and IGROVCDDP to CuSO_4_. B) ATP7A western blot. Open bars are IGROV-1, shaded bars are IGROVCDDP and striped bars indicate treatment with 0.67 µM cisplatin for 72 hours. Representative image shown. Graph shows quantitation of n = 4 biological repeats normalised to β-actin. C) CTR1 western blot. Representative image shown. Graph shows quantitation of n = 3 biological repeats normalised to β-actin. * Indicates significant difference from IGROV-1 p<0.05 student’s t-test. CTR1 confocal microscopy in D) IGROV-1 and E) IGROVCDDP cells. XY planes are shown for DAPI (blue), Golgi (red) and CTR1 (green), a merged image is also shown.

MRP2, a transporter which can efflux cisplatin conjugates, had decreased gene expression in IGROVCDDP (Table 2), but was not detectable by western blot in either cell line (data not shown). This suggests that there is no role of the platinum efflux transporter MRP2 in the platinum resistance of IGROVCDDP. Copper transporters can also play a role in platinum uptake (CTR1) and efflux (ATP7A and ATP7B) [17]. A decrease in CTR1 expression or increase in ATP7A or ATP7B could potentially mediate platinum resistance. The IGROVCDDP cells are 2.26 fold resistant to CuSO_4_ (Figure 2A) suggesting that copper metabolism may play some role in the mechanism of resistance. There was no significant change in the mRNA expression CTR1, ATP7A and ATP7B on the TLDA array (data not shown). ATP7A protein expression tended to increase in IGROVCDDP in response to cisplatin, but this change is not significant (Figure 2B). However, there was a significant decrease in CTR1 expression, in response to cisplatin drug treatment in the IGROVCDDP cells (Figure 2C). CTR1 is present in the cell membrane in IGROV-1 and shifts intracellularly to the cytoplasm in IGROVCDDP (Figure 2D, E). There is some association of CTR1 with the golgi in IGROVCDDP but the staining is consistent throughout the cytoplasm.

### Transporters as Biomarkers of the Platinum Accumulation Defect

**Figure 3:**
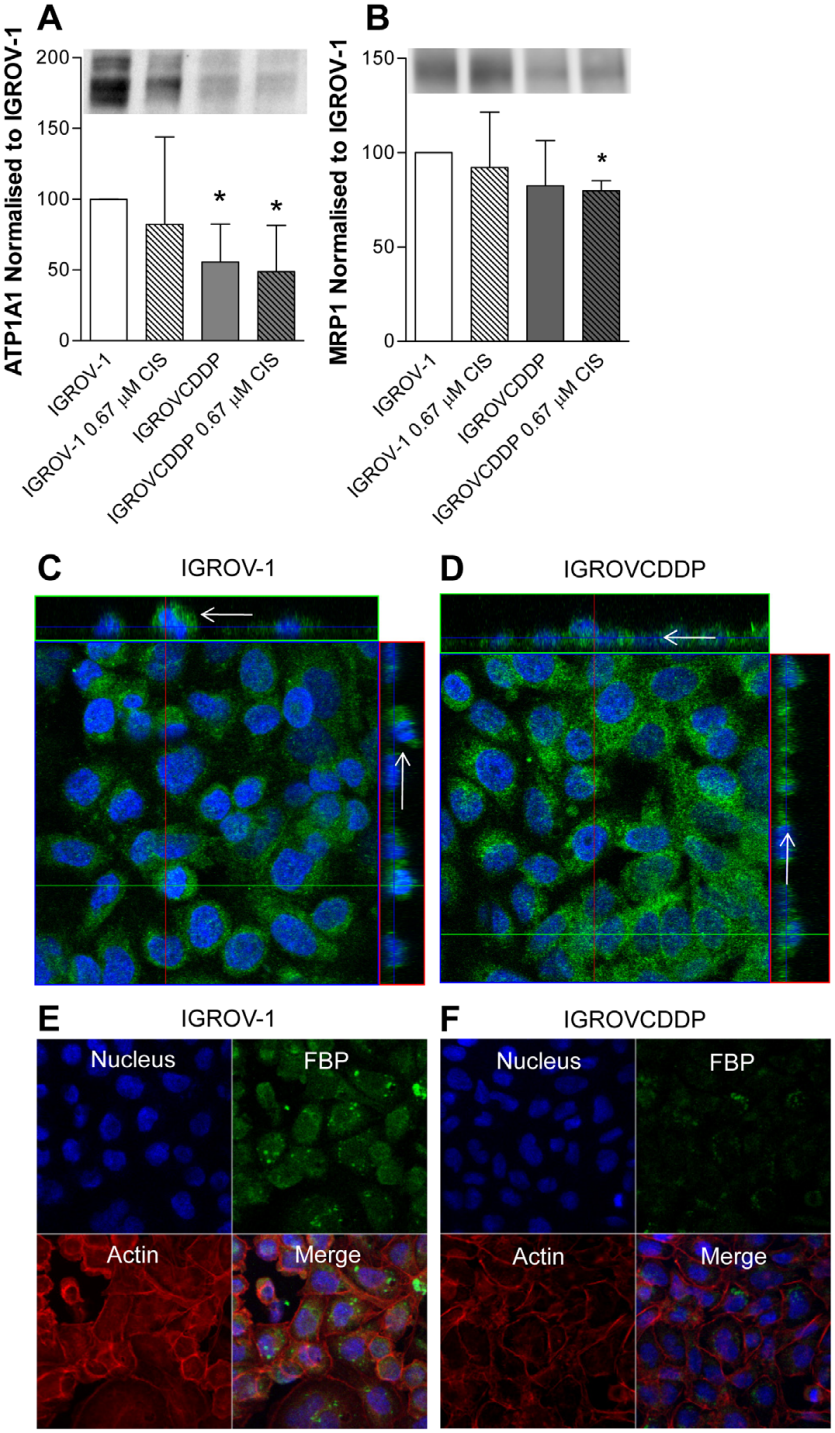
Biomarkers of platinum accumulation defect in IGROV-1 and IGROVCDDP cells. Open bars are IGROV-1, shaded bars are IGROVCDDP and striped bars indicate treatment with 0.67 µM cisplatin for 72 hours. A) ATP1A1 western blot. Representative image shown. Graph shows quantitation of n = 4 biological repeats normalised to β-actin. B) MRP1 western blot. Representative image shown. Graph shows quantitation of n = 3 biological repeats normalised to β-actin. * Indicates significant difference from IGROV-1 p<0.05 student’s t-test. MRP1 confocal microscopy in C) IGROV-1 and D) IGROVCDDP cells. Orthogonal images are shown for a merged image of DAPI (blue) and MRP1 (green), arrows on the side bars indicate the apical (IGROV-1) and basolateral location of MRP1 (IGROVCDDP). FBP confocal microscopy in E) IGROV-1 and F) IGROVCDDP cells. XY planes are shown for DAPI (blue), actin (red) and FBP (green), a merged image is also shown.

One of most significant differentially expressed genes in IGROVCDDP was a decrease in expression of the Na^+^/K^+^ pump (ATP1A1) (Table 2), which has previously been associated with platinum accumulation defects [18]. Cisplatin is not transported by ATP1A1 and altered membrane potential may play a role in the passive accumulation of the drug. There was a corresponding decrease in protein expression of ATP1A1 (Figure 3A) and also a sensitivity to treatment with the ATP1A1 inhibitor ouabain [19] (Table 3). However, when 0.01 nM ouabain was co-incubated in a cisplatin cytotoxicity assay rather than reversing the resistance in IGROVCDDP it decreased the IC_50_ of both the IGROV-1 and IGROVCDDP cells equally, the fold resistance between the two cell lines remained constant (Table 3). IGROVCDDP was not resistant to NaCl or KCl as single agents and the addition of 40 mM of these salts did not significantly alter cisplatin cytotoxicity (data not shown).

Previous research has shown decreased expression and an intracellular shift of membrane proteins MRP1 and FBP to be associated with a defect in platinum accumulation in cisplatin-resistant cell lines [20]. Therefore we examined MRP1 and FBP as potential biomarkers of a defect in platinum accumulation in IGROVCDDP. The IGROVCDDP cells have a decrease in mRNA expression of MRP1 (Table 2) as well as a small decrease in MRP1 protein expression in response to cisplatin treatment (Figure 3B). MRP1 distribution was examined by confocal microscopy (Figure 3C,D). Staining in both IGROV-1 and IGROVCDDP cell lines is evident in the cytoplasm and perinuclear region with some accumulations of MRP1 apparent. In IGROV-1 there is more MRP1 above and throughout the blue line on the orthogonal view indicating it is in the apical and mid-section in the cells. A majority of staining in IGROVCDDP is below the blue line it is basolaterally located. FBP is localised mainly adjacent to the nucleus within discrete sub-cellular vesicles; little cytoplasmic staining is evident (Figure 3E,F). There is no change in cellular distribution of FBP between IGROV-1 and IGROVCDDP, but a decrease in expression of FBP in IGROVCDDP is apparent from the confocal images.

**Figure 4:**
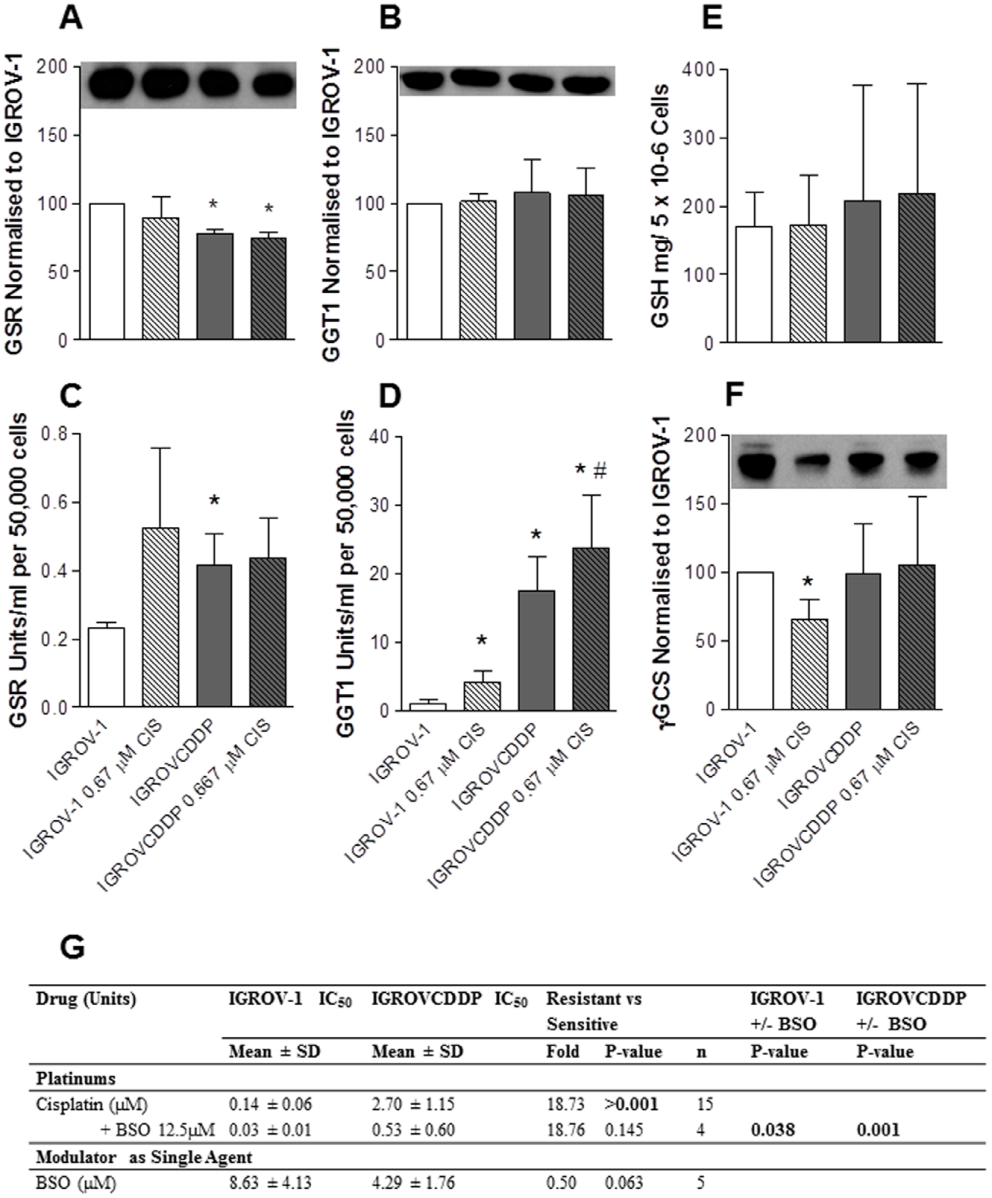
Glutathione pathway in IGROV-1 and IGROVCDDP cells. Open bars are IGROV-1, shaded bars are IGROVCDDP and striped bars indicate treatment with 0.67 µM cisplatin. A) Total intracellular glutathione. Graph shows n = 3 biological repeats normalised to cell number. B) γGCS western blot. Representative image shown. Graph shows quantitation of n = 4 biological repeats normalised to β-actin. C) GSR western blot. Representative image shown. Graph shows quantitation of n = 3 biological repeats normalised to β-actin. D) GSR enzyme assay. Graph shows n = 4 biological repeats normalised to cell number. E) GGT1 western blot. Representative image shown Graph shows quantitation of n = 3 biological repeats normalised to β-actin. E) GGT1 enzyme assay. Graph shows n = 4 biological repeats normalised to cell number. * Indicates significant difference from IGROV-1 p<0.05 student’s t-test. # Indicates significant difference from IGROVCDDP on the addition of cisplatin. G) Modulation of cisplatin cytotoxicity of IGROV-1 and IGROVCDDP with BSO.

### Platinum Resistance in IGROVCDDP is Associated with an Increase in Glutathione Recycling not Increased de novo Synthesis

The mRNA expression of glutathione reductase (GSR) and gamma glutamyl transpeptidase (GGT1) were both significantly increased in IGROVCDDP (Table 2). GSR functions to recycle oxidised glutathione within the cell [21], [22] and GGT1 recycles glutathione from outside the cell membrane [21], [22]. The protein expression of GSR and GGT1 were not increased in the IGROVCDDP cells, GSR was significantly decreased in IGROVCDDP and there was no change in GGT1. (Figure 4A and 4B). However, the enzyme activity of both GSR and GGT1 were significantly increased (Figure 4C and Figure 4D); suggesting that glutathione is being recycled more inside and from outside the cell.

**Figure 5:**
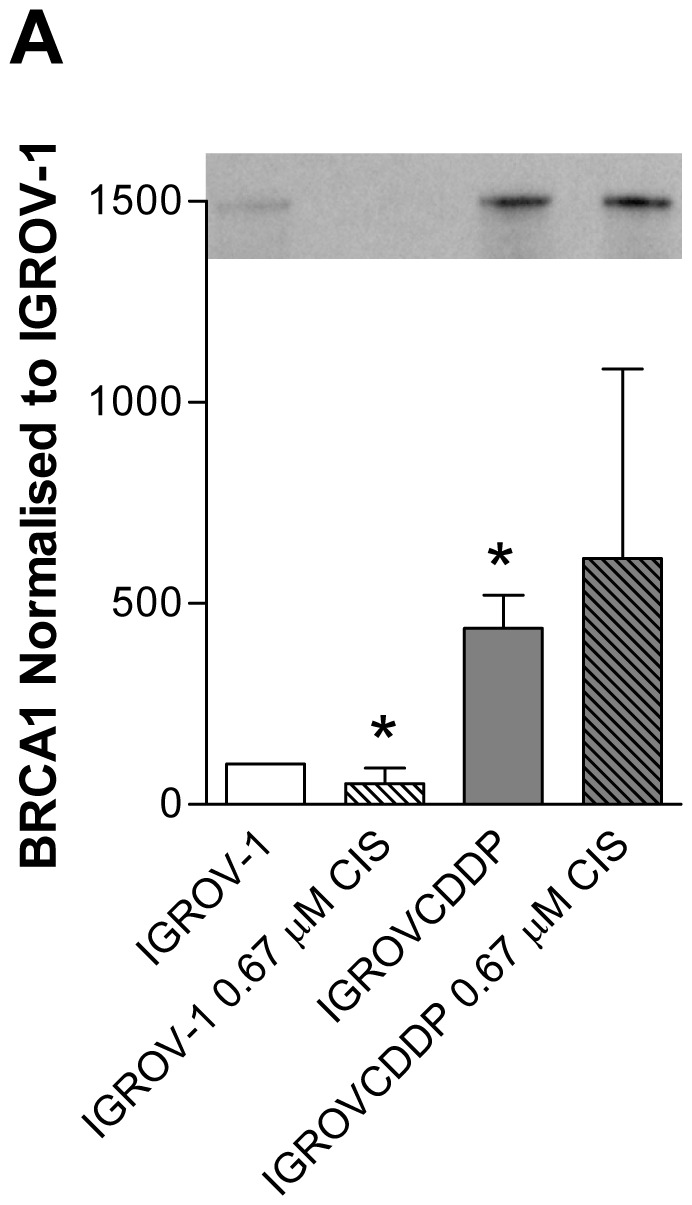
BRCA1 in IGROV-1 and IGROVCDDP cells. Open bars are IGROV-1, shaded bars are IGROVCDDP and striped bars indicate treatment with 0.67 µM cisplatin for 72 hours. A) BRCA1 western blot. Representative image shown. Graph shows quantitation of n = 4 biological repeats normalised to β-actin. * Indicates significant difference from IGROV-1 p<0.05 student’s t-test.

IGROVCDDP cells do not have higher levels of glutathione, and levels are not increased with low-level cisplatin treatment (Figure 4E). The levels of glutathione were also more variable in the IGROVCDDP cells. Treatment with 12.5 µM butathione sulfoximine (BSO), an inhibitor of γGCS [23] significantly decreased cellular glutathione in both the IGROV-1 and IGROVCDDP cells (data not shown). IGROV-1 and IGROVCDDP also have similar protein expression of γGCS and it is not upregulated in response to low-level cisplatin treatment (Figure 4F). BSO treatment also significantly sensitised both cell lines to cisplatin (Figure 4G). However, the effect was equivalent and the cisplatin fold resistance remained constant (18.76 fold). The IGROVCDDP cells tended to be more sensitive to BSO treatment alone in a cytotoxicity assay, however this was not significant (Figure 4G).

**Table 4:** Models of acquired cisplatin resistance which express P-glycoprotein.

Parent Cell Line	Cancer	Cisplatin IC50	ResistantCell Line	Cisplatin IC_50_	Cisplatin FoldResistance	P-glycoprotein				Reference
						↑DNA	↑mRNA	↑Protein	↑Activity	
LoVo	Colon	1.30±0.11 µg/ml	CP2.0	8.30±0.12 µg/ml	6.4	No	No	Yes	Yes	[26]
SKOV3	Ovarian	1.19±0.03 mM	SKOV3/CIS	4.42±0.3 mM	3.71	ND	ND	Yes	Yes	[27]
SKOV3	Ovarian	0.63±0.06 µM	SKOV3/DDP	9.92±0.34 µM	15.84	ND	ND	Yes	ND	[28]
SNU-601	Gastric	0.19 µg/ml	SNU-601/Cis2	9.2 µg/ml	48.42	ND	Yes	ND	Yes	[29]
			SNU-601/Cis10	>100 µg/ml	>526	ND	Yes	ND	No	
WS	Lymphoma (Rat)	900±30 nM	WR	7100±1000 nM	7.88	ND	ND	Yes	Yes	[30]

ND – Not Determined.

### IGROVCDDP has Increased BRCA1 Expression

Increased expression of the DNA repair gene BRCA1 has been previously associated with cisplatin resistance [24]. IGROVCDDP cells have increased mRNA (Table 2) and protein expression of BRCA1 (Figure 5A).

## Discussion

### P-gp Overexpression is Unusual in a Model of Acquired Cisplatin Resistance

Resistance to paclitaxel in IGROVCDDP cells is mediated by an overexpression of P-gp at the gene (Table 2) and protein level (Figure 1A). P-gp has been shown to be functionally active by cytotoxicity assays (Figure 1C) and epirubicin accumulation assays (Figure 1B). In contrast to other studies [25], short-term cisplatin treatment did not modulate P-gp protein expression, activity or taxane cytotoxicity in IGROVCDDP cells (Figure 1A, 1B, data not shown). It is unusual but not unprecedented to see a model of acquired cisplatin resistance overexpress P-gp (Table 4)[26]–[30]. This most likely represents a generalised stress response to long-term cisplatin treatment as cisplatin is not a P-gp substrate [13]. P-gp can be up-regulated as part of a response to increased reactive oxygen species (ROS) within a cell [31]. This may be why P-gp expression was induced in IGROVCDDP as ROS are also produced in response to cisplatin [32]. However, as the IGROVCDDP cells are grown without cisplatin in the media, there appears to be either another stimulus favouring the expression of P-gp or P-gp is providing a selective advantage to IGROVCDDP cells.

Many models of acquired drug resistance will have overexpression of an transporter which effluxes the drug that was used to develop the model. Colchicine, a P-gp substrate [33] selected for P-gp overexpression in KB-8-5-11 cells [34] and epirubicin, a MRP1 substrate [35] induced MRP1 expression in CCRF-CEM/E1000 [36]. However, methotrexate, fluorouracil, chlorambucil, cisplatin, and hydroxyurea have all been shown to transiently induce the expression of P-gp in K562 leukaemia cells when these drugs are not P-gp substrates [15]. It is then up to natural selection if the cells that transiently express P-gp have any other survival advantage and become part of the drug-resistant cell line. In some cisplatin-resistant models which overexpress P-gp, the P-gp has no survival advantage as is not functionally active (SNU-601/Cis10 - Table 4) [29]. Within cisplatin-resistant P-gp overexpressing cell lines there can also be heterogeneity; in SKOV3/CIS P-gp positive and negative populations were maintained after treatment with cisplatin, indicating that P-gp has no survival advantage for cisplatin treatment [27]. However, P-gp can have anti-apoptotic effects distinct from those associated with transport of cytotoxic drugs, and some may be mediated through efflux of pro-apoptotic glucosylceramide [37], [38]. It is also possible that some xenobiotic present in the FCS used to culture the cells could assist in maintaining the P-gp expression in IGROVCDDP.

IGROVCDDP is the only cisplatin-resistant model developed from IGROV-1 known to overexpress P-gp and consequently have a platinum/taxane-resistant phenotype. Cisplatin-resistant models IGROV-1/Pt0.5 and IGROV-1/Pt1 [39] have the inverse platinum/taxane-resistant phenotype. Other cisplatin-resistant IGROV-1 models have been developed (IGROV-R10, IGROV-1/CP) but they do not appear to have been examined for resistance to P-gp substrates or P-gp expression. However, P-gp has not been identified as differentially expressed by genomic or proteomic profiling [40]–[44].

### Platinum Resistance is Multifactorial

Platinum resistance in the IGROVCDDP cells is multifactorial and involves the glutathione pathway and decreased accumulation of drug. This could result either from a complex regulatory pathway which controls many different mechanisms for conferring resistance to cisplatin, or could reflect the fact that the cells were selected in multiple steps and could therefore have accumulated different mechanisms at each step.

IGROVCDDP cells are low-level resistant to CuSO_4_ suggesting a role of copper transport in platinum resistance (Figure 2A). The expression of uptake transporter CTR1 is reduced in IGROVCDDP in response to cisplatin drug treatment (Figure 2C), which may contribute to the decrease in platinum accumulation previously reported [3]. CTR1 also shifts from being membrane associated in IGROV-1 to cytoplasmic staining in IGROVCDDP (Figure 2D,E). The loss of a cisplatin uptake transporter from the cell membrane in IGROVCDDP is likely to be the cause of decreased cellular accumulation of platinum [3]. These results suggest that CTR1 needs to be examined for cellular localisation by immunohistochemistry (IHC), rather than by RT-PCR or Western blot to be useful as a biomarker of decreased accumulation of cisplatin. However, high levels of CTR1 as measured by RT-PCR and IHC have both been shown to be prognostic of sensitivity to frontline platinum chemotherapy in ovarian cancer [45]. It has been shown with other biomarkers of platinum resistance such as ERCC1 that mRNA expression can be prognostic even if mRNA expression does not directly correlate with the functional role of the protein [46]. ERCC1 is a DNA repair protein and the measurement of gene and protein expression does not strictly correlate with DNA repair activity. It could be similar with CTR1, gene and protein expression being prognostic independent of predicting protein function. By also examining protein localisation the sensitivity and specificity of CTR1 as a biomarker may be improved.

Our results show that while total cellular glutathione is not increased in IGROVCDDP (Figure 4E), the way glutathione is recycled in the cell is enhanced. Increased enzyme activity of GSR (Figure 4C) indicates oxidised glutathione is being recycled more efficiently to its reduced form. Increased enzyme activity of GGT1 (Figure 4D) indicates that GSH is being recovered from outside the cell and the precursor amino acids transported to be available for synthesis of new glutathione inside the cell.

### Biomarkers of Decreased Platinum Accumulation

Platinum accumulation defects mediated by decreased expression of ATP1A1 have been shown in H4-II-E/CDDP cisplatin resistant rat hepatoma cells [18]. The activity of ATP1A1 was previously associated with the mechanism of decreased cisplatin accumulation in IGROVCDDP as co-treatment with the inhibitor ouabain at a dose of 0.5 µg/mL reversed the decrease in accumulation [3]. Our cytotoxicity assays showed no reversal of platinum resistance when ouabain was added as an inhibitor (Table 3). The difference in results between studies may be the difference between a short-term high-dose ouabain treatment for an accumulation assay and a longer-term low-dose ouabain treatment in a cytotoxicity assay. IGROVCDDP cells are more sensitive to ouabain as a single agent (Table 3), consistent with the decrease in ATP1A1 protein (Figure 3A).

A mechanism has previously been described in cisplatin-resistant cell lines with platinum accumulation defects (KB-CP20 and 7404-CP20) in which surface expression of transporters is reduced, and some are overexpressed within cytoplasmic vesicles [47]. Protein expression of MRP1 is reduced in KB-CP20 and 7404-CP20 [48] and the protein is localised with the golgi rather than the cell membrane [20]. This is similar to what is observed in IGROVCDDP, decreased mRNA expression of MRP1 (Table 2), and decreased expression of MRP1 protein in response to cisplatin drug treatment (Figure 3B) and altered localisation within the cell (Figure 3C,D). KB-CP20, 7404-CP20 and IGROVCDDP cells are all resistant to the MRP1 substrate methotrexate due to the drug pump no longer being present on the cell surface, despite a decrease in protein expression [49]. Decreased expression and cytoplasmic localisation of FBP has also been associated with platinum accumulation defects in KB-CP20 and 7404-CP20 [49]. In IGROVCDDP the localisation of FBP does not change but the expression is decreased (Figure 4E,F). FBP is localised intracellularly in discrete vesicles near the nucleus rather than membrane associated. The altered localisation of MRP1 and FBP in IGROVCDDP is not extreme as what is seen in KB-CP20 and 7404-CP20 cells [20]. It is clear that to use a shift in MRP1 or FBP localisation as a biomarker of platinum accumulation defects the proteins must be strongly associated with the cell membrane in the parent cell line, in IGROV-1 this is not the case. Despite this caveat, a shift in MRP1 localisation appears to be more useful as a biomarker of a platinum accumulation defect than gene or protein expression. It has been shown that MRP1 gene [50] and protein expression [51] is not predictive platinum resistance in clinical ovarian samples, consistent with the results of this study. The localisation of MRP1 has not yet been examined in clinical samples.

### Potential Biomarkers of Platinum/taxane Cross Resistance Versus Inverse Resistance

The IGROVCDDP cells have increased mRNA (Table 2) and protein expression of BRCA1 (Figure 5A), which may contribute to platinum resistance through increased DNA repair. This result is particularly interesting as previously we have associated an increase in BRCA1 with the inverse resistance phenotype; platinum resistance and taxane sensitivity [24]. While an increase in BRCA1 may mediate taxane sensitivity in some models [52], [53] if there is an overriding mechanism of taxane resistance (such as P-gp) this effect is cancelled out. Therefore BRCA1 expression cannot be used as a molecular marker for platinum/taxane resistance status without also examining P-gp.

IGROV-1/Pt0.5 and IGROV-1/Pt1, platinum-resistant and taxane-sensitive cells, have increased cellular GSH and decreased GGT1 enzyme activity [39] which is the reverse pattern to that seen in the IGROVCDDP platinum/taxane-resistant cells. Further research is needed to determine if GGT1 activity could be used as biomarker which could predict whether a cisplatin-resistant cell line is resistant or sensitive to paclitaxel.

Decreased expression of FBP was also seen in IGROV1/Pt 0.5 and IGROV1/Pt 1. However, decreased FBP appeared to be an effect if cisplatin resistance rather than a cause of it as transfection with FBP cDNA did not cause cisplatin sensitivity [54]. The IGROVCDDP, IGROV1/Pt0.5 and IGROV1/Pt1 cells all have decreased expression of FBP, therefore the expression of FBP may be a useful biomarker of platinum resistance but cannot be used to differentiate between taxane sensitivity and resistance.

### Potential Treatment Strategies for Platinum/taxane Cross Resistant Ovarian Cancer

The only chemotherapy drug that IGROVCDDP was more sensitive to than IGROV-1 was 5-FU (Table 3). This suggests that 5-FU may be a suitable treatment for platinum/taxane resistant ovarian cancer. The changes in folate metabolism, as indicated by decreased expression of FBP (Figure 3E,F) may mediate this sensitivity to 5-FU. Several phase II clinical trials have examined capecitabine, a pro-drug of 5-FU, in platinum-resistant ovarian cancer. Platinum resistance was defined as progressive disease during or within 6 months of platinum treatment; patients in these studies had also received taxanes and therefore are most likely taxane resistant. The response rate of this population to capecitabine was poor 2.8–8.5% [55], [56]. This is similar to the response seen with single-agent oxaliplatin 7.6% [57] and is worse than retreatment with paclitaxel 35.3% [2]. Collateral sensitivity to 5-FU is not a universal feature of platinum/taxane resistant ovarian cancer or one would expect better results in the capecitabine clinical trials. The sensitivity of 5-FU in IGROVCDDP will be further investigated to determine its mechanism so biomarkers can be developed for use in the clinic.

### Conclusions

P-gp overexpression is rare in a model of acquired cisplatin resistance. In the IGROVCDDP cells P-gp causes taxane resistance and overrides any potential taxane sensitivity mediated by increased BRCA1 expression. Platinum resistance is multifactorial and is mediated by an increase in glutathione recycling and decreased accumulation of drug. The IGROVCDDP cells were sensitive to 5-FU and this class of chemotherapeutics warrants further preclinical research to determine if they are useful for the treatment of platinum/taxane resistant ovarian cancer.

## References

[1] Hennessy BT, Coleman RL, Markman M (2009). Ovarian cancer.. Lancet.

[2] Stordal B, Pavlakis N, Davey R (2007). A systematic review of platinum and taxane resistance from bench to clinic: an inverse relationship.. Cancer Treatment Reviews.

[3] Ma J, Maliepaard M, Kolker HJ, Verweij J, Schellens JH (1998). Abrogated energy-dependent uptake of cisplatin in a cisplatin-resistant subline of the human ovarian cancer cell line IGROV-1.. Cancer Chemotherapy & Pharmacology.

[4] Ma J, Maliepaard M, Nooter K, Boersma AW, Verweij J (1998). Synergistic cytotoxicity of cisplatin and topotecan or SN-38 in a panel of eight solid-tumor cell lines in vitro.. Cancer Chemotherapy & Pharmacology.

[5] Martin A, Clynes M (1993). Comparison of 5 microplate colorimetric assays for in vitro cytotoxicity testing and cell proliferation assays.. Cytotechnology.

[6] Gillet JP, Wang J, Calcagno AM, Green LJ, Varma S (2011). Clinical Relevance of Multidrug Resistance Gene Expression in Ovarian Serous Carcinoma Effusions.. Mol Pharmaceutics.

[7] Richard Simon, Amy Lam (2007). Analysis of Gene Expression Data Using BRB-Array Tools.. Cancer Informatics.

[8] Wall R, McMahon G, Crown J, Clynes M, O'Connor R (2007). Rapid and sensitive liquid chromatography-tandem mass spectrometry for the quantitation of epirubicin and identification of metabolites in biological samples.. Talanta.

[9] Suzukake K, Petro BJ, Vistica DT (1982). Reduction in glutathione content of L-PAM resistant L1210 Cells confers drug sensitivity.. Biochemical Pharmacology.

[10] Silber PM, Gandolfi AJ, Brendel K (1986). Adaptation of a gamma-glutamyl transpeptidase assay to microtiter plates.. Analytical Biochemistry.

[11] Cobbold C, Ponnambalam S, Francis MJ, Monaco AP (2002). Novel membrane traffic steps regulate the exocytosis of the Menkes disease ATPase.. Human Molecular Genetics.

[12] Hyafil F, Vergely C, Du Vignaud P, Grand-Perret T (1993). In Vitro and in Vivo Reversal of Multidrug Resistance by GF120918, an Acridonecarboxamide Derivative.. Cancer Research.

[13] Hamaguchi K, Godwin AK, Yakushiji M, O'Dwyer PJ, Ozols RF (1993). Cross-resistance to diverse drugs is associated with primary cisplatin resistance in ovarian cancer cell lines.. Cancer Research.

[14] Choudhuri S, Klaassen CD (2006). Structure, Function, Expression, Genomic Organization, and Single Nucleotide Polymorphisms of Human ABCB1 (MDR1), ABCC (MRP), and ABCG2 (BCRP) Efflux Transporters.. International Journal of Toxicology.

[15] Chaudhary PM, Roninson IB (1993). Induction of Multidrug Resistance in Human Cells by Transient Exposure to Different Chemotherapeutic Drugs.. J Natl Cancer Inst.

[16] Bates SE, Medina-Perez WY, Kohlhagen G, Antony S, Nadjem T (2004). ABCG2 Mediates Differential Resistance to SN-38 (7-Ethyl-10-hydroxycamptothecin) and Homocamptothecins.. Journal of Pharmacology and Experimental Therapeutics.

[17] Kuo MT, Chen HH, Song IS, Savaraj N, Ishikawa T (2007). The roles of copper transporters in cisplatin resistance.. Cancer & Metastasis Reviews.

[18] Kishimoto S, Kawazoe Y, Ikeno M, Saitoh M, Nakano Y (2006). Role of Na+, K+-ATPase alpha1 subunit in the intracellular accumulation of cisplatin.. Cancer Chemotherapy & Pharmacology.

[19] Takeyasu K, Tamkun MM, Renaud KJ, Fambrough DM (1988). Ouabain-sensitive (Na+ + K+)-ATPase activity expressed in mouse L cells by transfection with DNA encoding the alpha-subunit of an avian sodium pump.. Journal of Biological Chemistry.

[20] Liang XJ, Shen DW, Garfield S, Gottesman MM (2003). Mislocalization of membrane proteins associated with multidrug resistance in cisplatin-resistant cancer cell lines.. Cancer Research.

[21] Townsend DM, Tew KD, Tapiero H (2003). The importance of glutathione in human disease.. Biomedicine & Pharmacotherapy.

[22] Rahman Q, Abidi P, Afaq F, Schiffmann D, Mossman BT (1999). Glutathione redox system in oxidative lung injury.. Critical Reviews in Toxicology.

[23] Drew R, Miners JO (1984). The effects of buthionine sulphoximine (BSO) on glutathione depletion and xenobiotic biotransformation.. Biochemical Pharmacology.

[24] Stordal B, Davey R (2009). A systematic review of genes involved in the inverse resistance relationship between cisplatin and paclitaxel chemotherapy.. Current Cancer Drug Targets.

[25] Ihnat MA, Lariviere JP, Warren AJ, La Ronde N, Blaxall JR (1997). Suppression of P-glycoprotein expression and multidrug resistance by DNA cross-linking agents.. Clinical Cancer Research.

[26] Yang LY, Trujillo JM, Siciliano MJ, Kido Y, Siddik ZH (1993). Distinct P-glycoprotein expression in two subclones simultaneously selected from a human colon carcinoma cell line by cis-diamminedichloroplatinum (II).. International Journal of Cancer.

[27] Yang X, Page M (1995). P-glycoprotein expression in ovarian cancer cell line following treatment with cisplatin.. Oncology Research.

[28] Yang H, Zou W, Li Y, Chen B, Xin X (2007). Bridge linkage role played by CD98hc of anti-tumor drug resistance and cancer metastasis on cisplatin-resistant ovarian cancer cells.. Cancer Biology & Therapy.

[29] Xu H, Choi SM, An CS, Min YD, Kim KC (2005). Concentration-dependent collateral sensitivity of cisplatin-resistant gastric cancer cell sublines.. Biochemical & Biophysical Research Communications.

[30] Parekh H, Simpkins H (1996). Cross-resistance and collateral sensitivity to natural product drugs in cisplatin-sensitive and -resistant rat lymphoma and human ovarian carcinoma cells.. Cancer Chemotherapy & Pharmacology.

[31] Callaghan R, Crowley E, Potter S, Kerr ID (2008 March) Review/Drug Metabolism and Transport: P-glycoprotein: So Many Ways to Turn It On.. Journal of Clinical Pharmacology.

[32] Berndtsson M, Hägg M, Panaretakis T, Havelka AM, Shoshan MC (2007). Acute apoptosis by cisplatin requires induction of reactive oxygen species but is not associated with damage to nuclear DNA.. International Journal of Cancer.

[33] Kim RB (2002). Drugs as P-glycoprotein substrates, inhibitors, and inducers.. Drug Metabolism Reviews.

[34] Richert ND, Aldwin L, Nitecki D, Gottesman MM, Pastan I (1988). Stability and covalent modification of P-glycoprotein in multidrug-resistant KB cells.. Biochemistry.

[35] O'Connor R, O'Leary M, Ballot J, Collins CD, Kinsella P (2007). A phase I clinical and pharmacokinetic study of the multi-drug resistance protein-1 (MRP-1) inhibitor sulindac, in combination with epirubicin in patients with advanced cancer.. Cancer Chemotherapy & Pharmacology.

[36] Locke VL, Davey RA, Davey MW (1999). Altered drug sensitivity in response to idarubicin treatment in K562 human leukaemia cells.. British Journal of Haematology.

[37] Zhang X, Wu X, Li J, Sun Y, Gao P (2011). MDR1 (multidrug resistence 1) can regulate GCS (glucosylceramide synthase) in breast cancer cells.. J Surg Oncol.

[38] Lee WK, Torchalski B, Kohistani N, Thevenod F (2011). ABCB1 Protects Kidney Proximal Tubule Cells Against Cadmium-Induced Apoptosis: Roles of Cadmium and Ceramide Transport.. Toxicological Sciences.

[39] Perego P, Romanelli S, Carenini N, Magnani I, Leone R (1998). Ovarian cancer cisplatin-resistant cell lines: multiple changes including collateral sensitivity to Taxol.. Annals of Oncology.

[40] Poulain L, Lincet H, Duigou F, Deslandes E, Sichel F (1998). Acquisition of chemoresistance in a human ovarian carcinoma cell is linked to a defect in cell cycle control.. International Journal of Cancer.

[41] Sun B, Ranish JA, Utleg AG, White JT, Yan X (2007). Shotgun glycopeptide capture approach coupled with mass spectrometry for comprehensive glycoproteomics.. Molecular & cellular proteomics 6[1].

[42] Stewart JJ, White JT, Yan X, Collins S, Drescher CW (2006). Proteins associated with Cisplatin resistance in ovarian cancer cells identified by quantitative proteomic technology and integrated with mRNA expression levels.. Molecular & cellular proteomics 5[3].

[43] Cheng L, Lu W, Kulkarni B, Pejovic T, Yan X (2010). Analysis of chemotherapy response programs in ovarian cancers by the next-generation sequencing technologies.. Gynecologic Oncol 117[2].

[44] Le Moguen K, Lincet H, Deslandes E, Hubert-Roux M, Lange C (2006). Comparative proteomic analysis of cisplatin sensitive IGROV1 ovarian carcinoma cell line and its resistant counterpart IGROV1-R10.. Proteomics 6[19].

[45] Lee YY, Choi CH, Do IG, Song SY, Lee W (2011). Prognostic value of the copper transporters, CTR1 and CTR2, in patients with ovarian carcinoma receiving platinum-based chemotherapy. Department of Obstetrics and Gynecology, Samsung Medical Center, Sungkyunkwan University School of Medicine, Seoul 135–710, Republic of Korea.. Available.

[46] DeLoia JA, Bhagwat NR, Darcy KM, Strange M, Tian C (2011). Comparison of ERCC1/XPF genetic variation, mRNA and protein levels in women with advanced stage ovarian cancer treated with intraperitoneal platinum.. Gynecologic Oncology.

[47] Gottesman MM, Hall MD, Liang XJ, Bonetti A, Leone R, Muggia F, Howell SB (2009). Resistance to cisplatin results from multiple mechanisms in cancer cells..

[48] Shen DW, Goldenberg S, Pastan I, Gottesman MM (2000). Decreased accumulation of [14C]carboplatin in human cisplatin-resistant cells results from reduced energy-dependent uptake.. Journal of Cellular Physiology.

[49] Shen D, Pastan I, Gottesman MM (1998). Cross-resistance to methotrexate and metals in human cisplatin-resistant cell lines results from a pleiotropic defect in accumulation of these compounds associated with reduced plasma membrane binding proteins.. Cancer Research.

[50] Nakayama K, Kanzaki A, Ogawa K, Miyazaki K, Neamati N (2002). Copper-transporting P-type adenosine triphosphatase (ATP7B) as a cisplatin based chemoresistance marker in ovarian carcinoma: comparative analysis with expression of MDR1, MRP1, MRP2, LRP and BCRP..

[51] Lewandowicz GM, Britt P, Elgie AW, Williamson CJ, Coley HM (2002). Cellular glutathione content, in vitro chemoresponse, and the effect of BSO modulation in samples derived from patients with advanced ovarian cancer. Haematology Research, Pembury Hospital, Pembury, Tunbridge Wells, Kent TN2 4QJ, United Kingdom.. Available.

[52] Tassone P, Tagliaferri P, Perricelli A, Blotta S, Quaresima B (2003). BRCA1 expression modulates chemosensitivity of BRCA1-defective HCC1937 human breast cancer cells.. British Journal of Cancer.

[53] Quinn JE, Kennedy RD, Mullan PB, Gilmore PM, Carty M (2003). BRCA1 functions as a differential modulator of chemotherapy-induced apoptosis.. Cancer Research.

[54] Ottone F, Miotti S, Bottini C, Bagnoli M, Perego P (1997). Relationship between folate-binding protein expression and cisplatin sensitivity in ovarian carcinoma cell lines.. Br J Cancer 76[1].

[55] Pisano C, Morabito A, Sorio R, Breda E, Lauria R (2009). A phase II study of capecitabine in the treatment of ovarian cancer resistant or refractory to platinum therapy: a multicentre Italian trial in ovarian cancer (MITO-6) trial.. Cancer Chemother Pharmacol 64[5].

[56] Rischin D, Phillips KA, Friedlander M, Harnett P, Quinn M (2004). A phase II trial of capecitabine in heavily pre-treated platinum-resistant ovarian cancer.. Gynecologic Oncol 93[2].

[57] Stordal B, Pavlakis N, Davey R (2007). Oxaliplatin for the treatment of cisplatin-resistant cancer: A systematic review.. Cancer Treatment Reviews.

